# The Graded Priming Effect of Semantic Radical on Chinese Character Recognition

**DOI:** 10.3389/fpsyg.2021.611066

**Published:** 2021-02-23

**Authors:** Xiuhong Tong, Mengdi Xu, Jing Zhao, Liyan Yu

**Affiliations:** ^1^Department of Psychology, The Education University of Hong Kong, Hong Kong, Hong Kong; ^2^Institute of Psychological Sciences, Hangzhou Normal University, Hangzhou, China

**Keywords:** semantic priming, Chinese character recognition, graded priming effect, sematic relatedness, adults

## Abstract

This study used priming paradigm with lexical decision task to examine the effects of different levels of semantic relatedness on the identification of Chinese phonetic–semantic compound characters. Unlike previous studies that simply classify Chinese compound characters as semantically transparent or opaque, we categorize the semantic relatedness between semantic radicals (i.e., prime) and the target characters containing them into five levels: highly related (i.e., high condition; e.g., prime ± vs. target 地), moderately related (i.e., moderate condition; e.g., prime ± vs. target 场), minimally related (i.e., minimal condition; e.g., prime ± vs. target 塔), unrelated but sharing the semantic radical (i.e., form-only condition; e.g., prime ± vs. target 坏), and unrelated without sharing the semantic radical (i.e., control condition; e.g., prime ± vs. target 涌). Moreover, three stimulus-onset asynchrony (SOA)s (i.e., 57, 140, and 243 ms) were used in this study to dissociate the radical- and character-level semantic priming effects. Results revealed a graded priming effect of the semantic radical on character recognition in Chinese readers for all SOAs. More specifically, the facilitative effect of the semantic radical on character processing was most evident for the high condition, followed by the minimal, form-only, and control conditions. This suggests a graded priming effect of the semantic radical on character identification.

## Introduction

Visual word recognition involves the complex cognitive processing of orthographic, phonological, and semantic information (e.g., Seidenberg and McClelland, 1989; Tong and McBride, 2018), which varies across language systems. Phonological processing is the default route to visual word recognition in alphabetic languages, whereas semantic processing appears to be more important in Chinese character recognition given the logographic nature of the Chinese writing system (e.g., Wang et al., 2017; Tong and McBride, 2018). Chinese is described as a morphosyllabic language, in which the basic visual unit, i.e., the character, simultaneously represents a syllable and, usually, a morpheme (the smallest unit of meaning in a language). The majority of Chinese characters (over 80%) are semantic–phonetic compound characters containing submorphemic units, namely semantic and phonetic radicals (Zhou, 1978). Phonetic and semantic radicals have both positional and functional regularities. Phonetic radicals, usually located on the right side of a character, provide certain clues regarding the sound of the character. Semantic radicals, usually occupying the left-hand position in a character, sometimes indicate the meaning of the character. These are called semantically transparent characters (i.e., those whose meanings are semantically related to the semantic radicals). There are also semantically opaque characters whose meanings are unrelated to the semantic radicals. Around 65% of compound characters are semantically transparent such that the meaning of the whole character is semantically related to its semantic radical (Fan et al., 1984).

Contemporary models of visual word recognition have specified the role of radicals in Chinese character recognition hypothesizing that radical representation is activated parallel to character-level processing, particularly for radicals with their own meanings and sounds in Chinese (Zhou and Marslen-Wilson, 1999). Recently, Tong and McBride (2018) proposed a new developmental model, i.e., the psycholexical space mapping model (PSLM), to illustrate how sublexical and lexical information are involved in Chinese character reading. The PSLM model posits that: (1) phonological, semantic, and positional information at both lexical and sublexical levels interactively determine the process of Chinese character recognition; (2) the representation of phonetic and semantic radicals in mental lexicon is graded and dynamic; and (3) statistical learning is a powerful mechanism underlying semantic and phonetic regularity learning in children. The PSLM model also suggests a developmental difference in the use of lexical and sublexical information in word recognition at different developmental stages of reading (Tong and McBride, 2018).

A number of studies using different paradigms have supported the theoretical hypothesis that radicals in Chinese are activated and important in Chinese character recognition (Taft et al., 1999). However, most prior studies have conceptualized the semantic relatedness between semantic radicals and characters into two types: an absolute concept (i.e., semantically transparent characters) and a dichotomous concept (i.e., semantically opaque characters). According to the PSLM model, the regularity between radicals and the characters containing them is a continuous variable rather than a bipolarized variable (i.e., regular vs. irregular for phonetic radicals; transparent vs. opaque for semantic radicals). Thus, an increasing priming effect of semantic relatedness is expected from no semantic relationship (e.g., 坏-±) to a minimal (e.g., 塔-±), moderate (e.g., 场-±), and high semantic relationship (e.g., 地 -±) in primed character decision-making. However, no prior studies have explored how the variations in semantic relatedness between semantic radicals and the characters containing them influence Chinese character recognition. In this study, we used priming paradigm with lexical decision task to examine the effects of different levels of semantic relatedness on the identification of Chinese phonetic–semantic compound characters.

Various studies with different behavioral paradigms have shown facilitative effects of the semantic relatedness of radicals on character recognition (e.g., Zhou and Marslen-Wilson, 1999; Chen and Weekes, 2004; Williams and Bever, 2010). Using priming paradigm with a character decision task in two experiments, Feldman and Siok (1999) examined the activation of semantic radicals in Chinese character recognition by manipulating semantic relatedness between primes and targets. There were four types of primes: semantically related prime with shared semantic radical (e.g., 评 vs. 论), semantically related prime without shared semantic radical (e.g., 述 vs. 论), semantically opaque prime (e.g., 诸 vs. 论), and control (e.g., 竿 vs. 论). In experiment 1, the stimulus-onset asynchrony (SOA) was 243 ms between primes and targets. In experiment 2, there was a lag (i.e., 10 items) between primes and targets. The authors reported a semantic priming effect for semantically related primes for both shared and unshared semantic radicals compared with the control condition. However, an inhibition effect was revealed in the semantically opaque condition in experiment 1. Semantic priming effect was found only in the semantic transparent condition when the prime and target were separated by 10 items. Feldman and Siok (1999) showed that the semantic radical is essential to Chinese character recognition because the semantic priming effect of the semantic radical was independent of the orthographic similarity or semantics of the whole character. Williams and Bever (2010) reported that, in a semantic categorization task, semantically transparent compound characters with embedded semantic radicals (e.g., 狼 *wolf* and its semantic radical 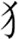 indicating an animal-related concept) were more quickly and accurately categorized as an animal than semantically opaque compound characters (e.g., 获 *capture* and 虎 *tiger*).

One limitation of earlier studies is the categorization of the semantic relatedness between the semantic radical and the target character into two types, that is, semantically transparent and semantically opaque. As described above, the semantic relatedness between targets and semantic radicals, particularly those semantic radicals that can stand alone with their own sound and meaning, can be categorized into more than two types. Additionally, most past studies used compound characters to serve as primes in the priming paradigm, which may have led to the confounding effect of the meaning of the whole word on the targets. For example, if the compound character “林” is used as the prime, not only is the meaning of the semantic radical “木” activated to influence the processing of the target “杆” but so is the meaning of the whole character “林.” To exclude the influence of the whole compound character, the present study used semantic radicals that can stand alone as primes.

This study examined how semantic relatedness influences semantic–phonetic compound character processing. The semantic relatedness between semantic radicals (i.e., prime) and the characters containing them was classified into five types: (1) highly related (i.e., high condition; e.g., prime ± vs. target 地), moderately related (i.e., moderate condition; e.g., prime ± vs. target 场), minimally related (i.e., minimal condition; e.g., prime ± vs. target 塔), unrelated but sharing the semantic radical (i.e., form-only condition; e.g., prime ± vs. target 坏), and unrelated without sharing the semantic radical (i.e., control condition; e.g., prime ± vs. target 涌). The first three prime types were semantically related to the targets but differed in terms of the semantic transparency between the primes and the targets. The form-only condition was used to control the contribution of orthographic similarity on the effect of semantic transparency of a radical for compound characters containing the semantic radical. The control condition was a baseline against which to evaluate the effects of the other four types of primes.

In addition, past studies have shown that the activations of orthographic, phonological, and semantic information vary with different SOAs (Zhou and Marslen-Wilson, 2000). For example, orthographic information was more likely to facilitate target recognition at the earliest processing stage when the targets and prime shared similarities in form (e.g., Stolz and Feldman, 1995), but the facilitative effect of morphological information on the recognition of targets was observed in both short SOAs and longer lags (Zhang and Zhang, 2016). We thus used different SOAs in this study based on past studies [i.e., 57 ms (Perfetti and Tan, 1998; Zhou and Marslen-Wilson, 2000), 140 ms (Perfetti and Zhang, 1995), and 243 ms (Tan and Perfetti, 1997)] to dissociate radical- and character-level priming effects. We hypothesized that the semantic priming effects would be observed across experimental conditions in all three SOAs if the semantic priming effects reflect the semantic radical-level facilitative effect. That is, we hypothesized that if variations in semantic relatedness between the prime and the target were present, then differences in target response latencies or accuracy should be observed across conditions, with the shortest reaction time and highest accuracy rate for the high condition, followed by the moderate, minimal, form-only, and control conditions.

## Methods

### Participants

A total of 84 right-handed participants were recruited from a university in China. All participants were native Chinese speakers with normal or corrected-to-normal vision. None of them reported any history of neurological or psychiatric disorders. Four participants were excluded from the final data analysis: one subject responded to < 30% of the trials, another had a high error rate (>20%), and two subjects appeared drowsy during the experiment. Eighty subjects (*M* = 20.10, SD = 2.07, range 18–26, 70 females) were included in the analysis: 26 of them under the condition of 57 ms SOA, 27 under the condition of 140 ms SOA, and 27 under the condition of 243 ms SOA. Ethical approval for this study was obtained from the University Ethics Committee. Participants received corresponding remuneration after completing the experiment.

### Materials and Design

The priming paradigm was adopted with a single-factor design (i.e., the semantic relatedness between the semantic radical and the character containing the semantic radical). The semantic radicals were used as primes, and the characters containing them were the targets. There were five types of primes: high, moderate, minimal, form-only, and control.

The experimental material was composed of characters and noncharacters. All targets used in this study were compound characters with stand-alone semantic and phonetic radicals selected from a recognized and widely used online modern Chinese database (Chinese Text Computing at http://lingua.mtsu.edu/chinese-computing/, by Dr. Jun Da) (Li et al., 2011; Wang et al., 2017). Thirty-one semantic radicals (e.g., 舟, 土, 车) were chosen, each forming five characters, so that a total of 155 Chinese characters were used. Eighty characters were left–right structure, 70 were top–bottom, and five were half-surrounded. One-hundred and fifty-five noncharacters served as fillers and were shown to all the participants. To avoid the practice effect, all noncharacter targets were constructed either by changing one or more strokes of real characters or by combining two components that did not co-occur. The proportion of radical-sharing items for characters and noncharacters was the same. Thus, in noncharacter trials, there were 31 radicals, with each radical used five times. Moreover, we counterbalanced the prime–target pairings by presenting the materials in two different versions. In version 1, the first half of the trials used primes to prime the targets, while the second half used targets to prime the primes. Version 2 was the reverse.

Fifteen native Chinese speakers who did not participate in the lexical decision experiment were required to evaluate the semantic relationships between semantic radicals and the whole characters using a seven-point Likert scale, with 1 indicating highly semantically unrelated and 7 indicating highly semantically related. A single-factor repeated measurement ANOVA was performed with semantic relatedness as the within factor (five levels: high, moderate, minimal, form-only, and control). The result showed that there were significant differences across five conditions, *F*(4, 11) = 203.51, *p* < 0.001. *Post hoc* multiple comparisons found that the semantic relatedness score for the high condition was significantly higher than that for the other four conditions (*p*s < 0.001); the semantic relatedness score for the moderate condition was significantly higher than that for the minimal, the form-only, and the control conditions (*p*s < 0.001); the semantic relatedness score for the minimal condition was significantly higher than that for the form-only and the control conditions (*p*s < 0.001); and the semantic relatedness score for the form-only condition was significantly higher than that for the control condition (*p* < 0.001).

As each character has one frequency, the frequency was considered as a continuous variable in the present study. As the skewness values of the five conditions were bigger than 1 (McNeese, 2008), so log transformation was done before the ANOVA analysis for the frequency. After the log transformation, the statistics of skewness were smaller than 1. The final ANOVA analysis showed that there was no significant difference in Chinese character frequency across different levels, *F*(4, 150) = 1.68, *p* = 0.16. As the difference in character frequency was not significant, the frequency was not considered as a control variable in the ANOVA analysis and linear mixed model analysis for the reaction time and accuracy rate. Moreover, there was no significant difference in the number of strokes between different levels of characters, *F*(4, 150) = 0.39, *p* = 0.82. The stimuli characteristics for each condition are shown in Table 1.

**Table 1:** Examples and characteristics of stimuli for each experimental condition.

	**High**	**Moderate**	**Minimal**	**Form-only**	**Control**
Radical	± (earth)	± (earth)	± (earth)	± (earth)	± (earth)
Character	地 (ground)	场 (field)	塔 (tower)	坏 (bad)	涌 (stream)
Stroke	10.06 (2.63)	9.56 (2.76)	10.19 (3.28)	10.52 (3.55)	9.87 (2.38)
Frequency^a^	9.71 (1.73)	9.82 (1.25)	9.41 (1.50)	10.45 (1.65)	9.83 (1.91)
Semantic rating score	6.07 (0.65)	5.01 (0.71)	3.72 (1.16)	2.47 (0.71)	1.58 (0.41)

a *The mean and standard deviation after log transformation; frequency = LN (initial frequency)*.

### Procedure

**Figure 1:**
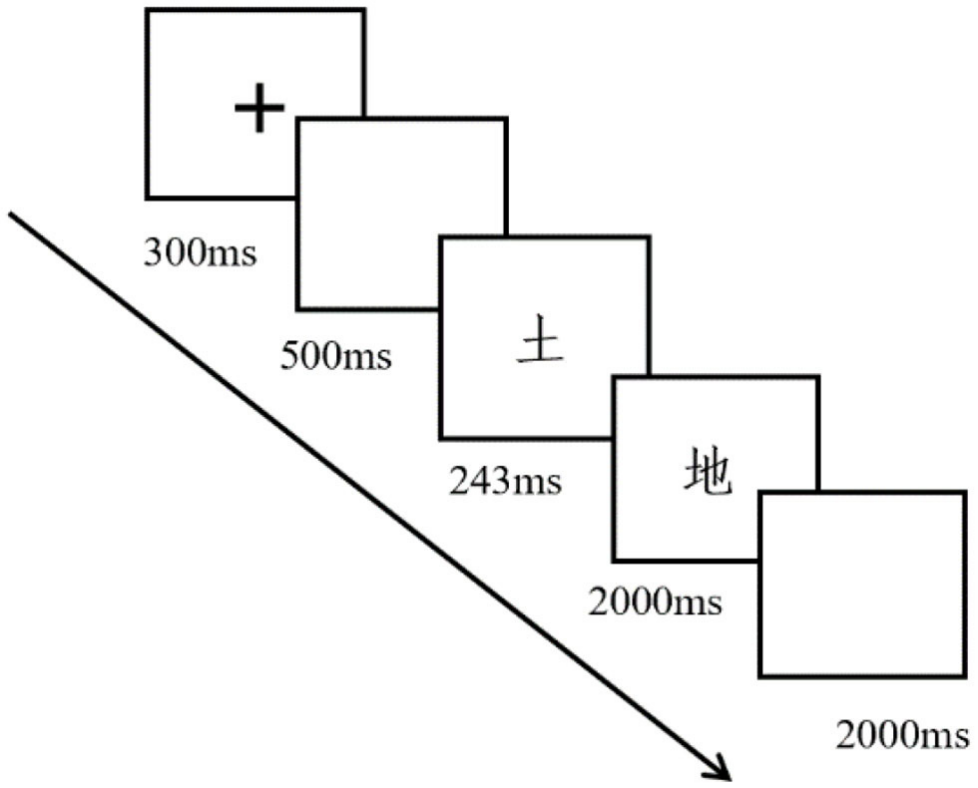
The experimental procedure.

Each participant was tested individually in a psychology department lab at the university. All participants were required to read and sign a consent form, which was approved by the University Ethical Committee. During the experiment, each participant was seated in a comfortable chair 70 cm from a computer monitor. A practice block was used to familiarize the participants with the task. Using the E-Prime software package (Psychology Software Tools, Pittsburgh, PA), stimuli were presented in Kai typeface in the center of the computer screen, with a visual angle of 1.70° vertical and 1.70° horizontal. As shown in Figure 1, for each trial, a fixation “+” lasting 300 ms was first presented in the center of the screen. Next, a prime lasting 243 ms appeared in the same place as the fixation “+,” followed immediately by the target for a duration of 2,000 ms. After each stimulus presentation, a blank screen appeared for 2,000 ms as an interstimulus interval (ISI). Participants were required to judge whether the targets (i.e., the second item in a continued presentation on the screen) were real words by pressing the correct buttons on the keyboard (i.e., the “J” key for a “yes” response and the “F” key for a “no” response) within 2,000 ms. The experiment used a pseudo-random design. Each participant took approximately 30 min to complete the whole experiment (for the experiment procedure, see Figure 1).

## Results

**Figure 2:**
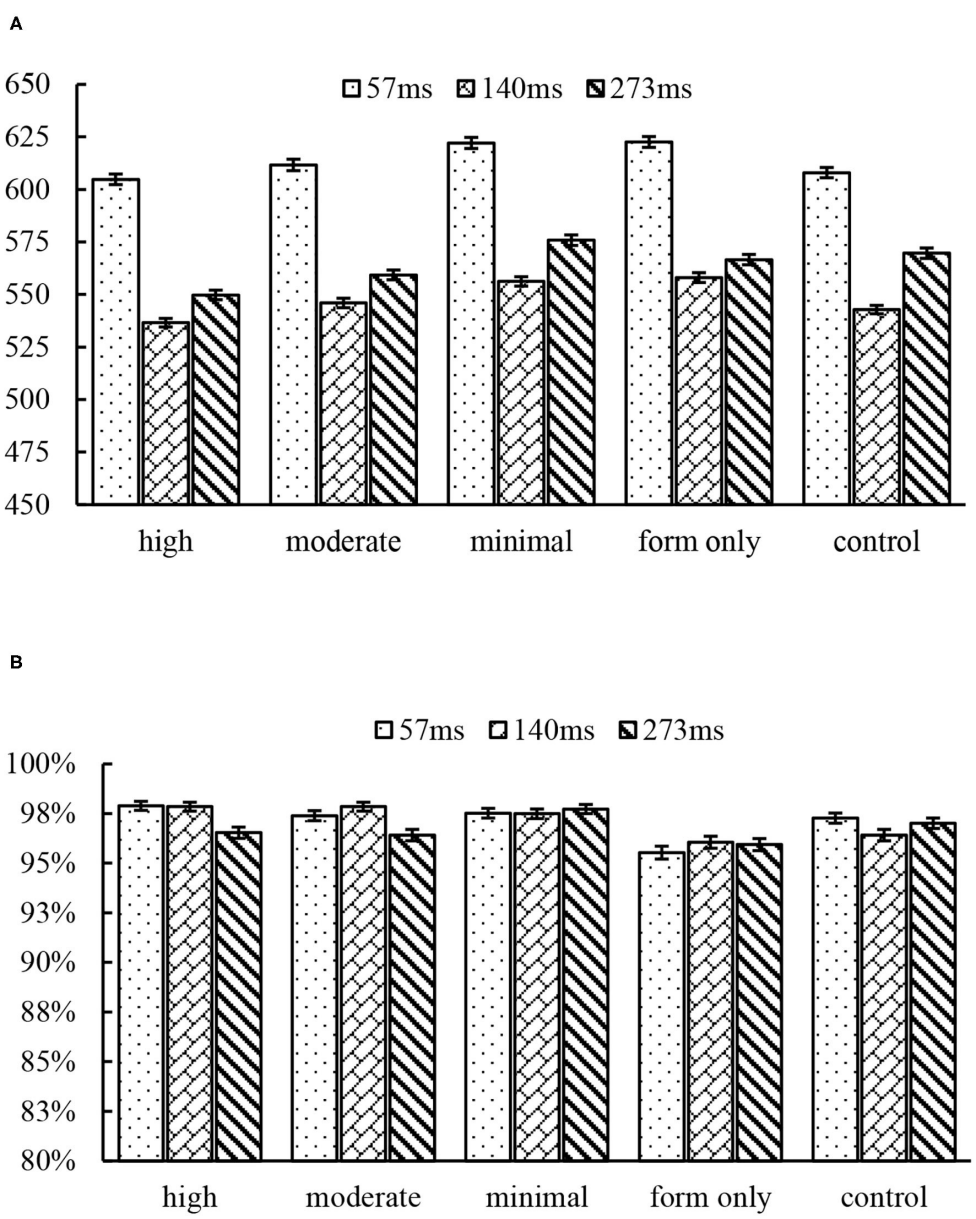
The mean reaction time (ms) **(A)** and accuracy **(B)** by conditions and SOA. Error bars indicate standard error.

Data were analyzed only for real characters. Data that were more extreme than three standard deviations were excluded from the analysis. Table 2 shows the descriptive statistics of reaction times and accuracy, and Figure 2 illustrates the reaction times and accuracy of the five conditions.

**Table 2:** Means and standard deviations (SDs) of reaction time (RT) and accuracy (Acc) across three SOAs and five conditions.

**SOA (ms)**	**Prime type**				
	**High**	**Moderate**	**Minimal**	**Form-only**	**Control**
**Reaction time (ms)**					
57	604.81 (155.82)	611.67 (167.71)	622.14 (162.76)	622.61 (159.17)	607.99 (151.14)
140	536.61 (128.03)	545.97 (141.52)	556.24 (140.17)	558.07 (147.93)	542.84 (126.83)
273	549.79 (144.64)	559.33 (146.31)	575.86 (157.97)	566.60 (156.02)	569.68 (156.22)
**Accuracy rate (%)**					
57	97.89 (14.38)	97.39 (15.94)	97.52 (15.57)	95.53 (20.67)	97.27 (16.30)
140	97.85 (14.51)	97.85 (14.51)	97.49 (15.65)	96.06 (19.47)	96.42 (18.60)
273	96.54 (18.3)	96.42 (18.60)	97.73 (14.90)	95.94 (19.75)	97.01 (17.03)

*Values in parentheses are standard deviations*.

We used a linear mixed effects (lme) model to analyze the data in R (Version 4.02; R Core Team, 2017). The linear mixed models were constructed with the glmer function from the lme4 package (Version 1.1-7; Bates et al., 2014). In the linear mixed models, the five conditions (i.e., high, moderate, minimal, form-only, and control) were included as the fixed effect. The by-participant and by-item random intercepts were considered, but not random slope. The ANOVA function was then used to calculate the significance across conditions. Finally, a multiple comparison procedure was performed using the glht function in the “multcomp package” (Hothorn et al., 2016), and the Holm–Bonferroni method was used to adjust the results of this test. For the results of model fit, we reported the regression coefficients (*b*, effects relative to the intercept), standard errors (SE), and *p* values.

### 57 ms SOA

Results of the model analysis suggest that semantic priming effects were found across conditions in both reaction time and accuracy. More specifically, the reaction time for the high condition was significantly shorter than that for the minimal condition (*b* = 19.11, SE = 6.31, *p* < 0.05) and the form-only condition (*b* = 18.93, SE = 6.36, *p* < 0.05). The accuracy rate for the form-only condition was significantly lower than that for the high condition (*b* = 0.02, SE = 0.01, *p* < 0.05).

### 140 ms SOA

Results of the model analysis suggest that semantic priming effects were found across conditions, but only in reaction time, and not accuracy. The reaction time for the high condition was significantly shorter than that for the minimal condition (*b* = 18.56, SE = 5.27, *p* < 0.01) and the form-only condition (*b* = 20.33, SE = 5.29, *p* < 0.01). The control condition was significantly shorter than the form-only condition (*b* = 15.03, SE = 5.31, *p* < 0.05).

### 243 ms SOA

Results for the 243-ms SOA are the same as in experiment 1. That is, the semantic priming effects were found across conditions in reaction time but not accuracy (all *p*s > 0.38). The reaction time for the high condition was significantly shorter than that for the minimal condition (*b* = 25.49, SE = 6.07, *p* < 0.001) and the control condition (*b* = 18.89, SE = 6.07, *p* < 0.05).

It is noted that a slightly longer reaction time was observed in the minimal condition than in the control conditions under all SOAs, suggesting a tendency of an inhibition effect, and there is a tendency for an inhibition effect in the form-only condition under 57 and 140 ms SOAs relative to the control condition. However, the inhibition effect did not reach significance at the statistical level.

## Discussion

The present study systematically examined the role of the semantic radical in Chinese character recognition by manipulating the semantic relatedness between the semantic radical and the character containing it. In almost all previous studies, semantic relatedness is categorized as two types: semantically related vs. semantically unrelated. However, in the present study, semantic relatedness was classified as highly semantically related, moderately related, minimally related, and form-related. A control condition was also included. Results demonstrate that, regardless of SOA, the facilitative effect of the semantic radical on the processing of characters is most evident for the high condition, followed by the minimal, form-only, and control conditions.

The findings of the present study suggest that the semantic radical is activated during Chinese visual character recognition, which is consistent with some prior studies showing the priming effect of the semantic radical on Chinese visual word recognition (Feldman and Siok, 1999). Our findings support most psycholinguistic models of visual character recognition that explicitly emphasize the role of sublexical radicals in Chinese character recognition. For example, the distributed connectionist account postulates that radical representation, entailing orthographic, phonological, and semantic constituents, is activated in tandem with character-level processing (Zhou and Marslen-Wilson, 1999), while the lexical constituency model (Perfetti et al., 2005) further clarifies that radicals are activated only as independent characters defined by orthographic, phonological, and semantic constituents. Together with these past studies, the findings of our study suggest that semantic radicals, as a component of Chinese characters, are important in character recognition. The semantic radical can facilitate the recognition of the character when the meaning of the semantic radical matches the meaning of the character containing it (e.g., Feldman and Siok, 1999). However, when the meaning of the semantic radical does not correspond to the character containing it, no facilitative effect is expected. More importantly, the present study shows that the facilitative priming effect of semantic radicals on characters is not bipolarized, but changes linearly (Tong and McBride, 2018). For example, we found that the facilitative effect of the semantic radical on the processing of characters is most evident for the highly related condition, followed by the moderately and minimally related conditions. Our findings thus provide empirical support for the hypothesis proposed by the PLSM model that the semantic relatedness between the semantic radical and the character containing it is graded and is a continuous variable that can be quantified (Tong and McBride, 2018).

Notably, this study used stand-alone semantic radicals. It is argued that the semantic priming effect observed in long SOA (i.e., 243 ms in this study) might be attributed to the activation of meaning at the lexical level (i.e., the whole character semantic relatedness) rather than at the sublexcial level (i.e., the semantic radical-level relatedness). For example, the facilitative effect of the prime “±” on the target “地 ” might be due to the activation of the meaning of “地 ” rather than its semantic radical “±.” If so, then the semantic priming effects of stand-alone semantic radicals on targets that contain them are not expected with an SOA under 100 ms. This is in accord with the orthographic similarity account in which orthographic similarity, but not semantics, facilitates target recognition (e.g., Perfetti and Zhang, 1995). However, the findings observed in short SOAs (i.e., 57 and 140 ms) in this study revealed the same priming effects in both short (i.e., 57 ms) and long (i.e., 140 and 243 ms) SOAs. Our findings support Feldman and Siok's (1999) argument that orthographic similarity and whole character relatedness were not sufficient to account for the semantic priming effects in this study, suggesting that the activation of semantic radicals can also facilitate visual word recognition.

Additionally, an inhibition effect appears to show in the minimally related condition under all SOAs, and there is a tendency for an inhibition effect in the form-only condition under 57 and 140 ms SOAs. These findings are inconsistent with prior studies showing an orthographic facilitative effect of the prime on the target when they share orthographic similarities within the 100-ms SOA on the character decision task (Tan and Peng, 1991). However, our findings are in line with Feldman and Siok's (1999) study reporting an inhibition effect when primes and targets are orthographically similar under a 243-ms SOA. Feldman and Siok (1999) argued that the inhibition effect might be caused by the inconsistent meaning of the semantic radicals relative to the whole character in an opaque prime. For example, in the present study, the meaning of the semantic radical “±” is “earth,” but the meaning of the whole character “坏” is “bad” in the form-only condition. This indicates that the meaning of the target does not correspond to the meaning of the semantic radical. This inconsistency may cause the meaning of the radical to become inhibited some time after the initial activation of the prime (Feldman and Siok, 1999).

The observed patterns of the semantic radical priming effect on Chinese characters are consistent with a study on the word length effect on Chinese character recognition reported in a Megastudy of Lexical Decision in Simplified Chinese (MELD-SCH) (Tsang et al., 2018). Similar to the present study, a U-shape was revealed for the influence of Chinese character length on Chinese character recognition with the recognition of one-character and four-character words slower than two- and three-character words. Our study together with that of Tsang et al. (2018) suggests that theoretical models and empirical studies should consider the dynamic features of the Chinese language at each linguistic level (i.e., orthographic, phonological, and semantic). However, we must be cautious when making a conclusion on the graded feature of semantic radicals in Chinese character recognition because only stand-alone semantic radicals were included in this study. In Chinese, there are also some semantic radicals that cannot stand alone. For example, the semantic radical “扌” can only be used with other radicals or strokes to form new characters (e.g., 把). Both types of radicals were found to be processed differently in an event-related potential (ERP) study with a masked priming character decision task (Zou et al., 2019). Specifically, compared with noncharacter semantic radicals, a less widely distributed P200 and earlier N400 (300 ms) were elicited for stand-alone character radicals. A late positive complex component was also elicited only for character semantic radicals. The authors argued that the differences in P200 between the two types of radicals might occur for two possible reasons: (1) the less distinctive orthographic features of noncharacter radicals compared with character radicals and (2) the unmatched visual features, such as the difference in the number of strokes and repetitions, between the two types of radicals. The different N400 effect might be due to the fact that character radicals have both radical and character representations, which lead to a stronger link between radicals and meanings than noncharacter radicals, which only have radical representation. One possible extension of the present study is to include both stand-alone semantic radicals and noncharacter radicals in one study to examine whether both types of semantic radicals are graded and can be evaluated quantitatively in terms of semantic relatedness with characters containing them. Additionally, as we noted earlier, the semantic radical priming effects across experimental conditions might be due to the activation of the lexical level rather than the sublexical level (i.e., semantic radicals) although the results of the present study showed the semantic priming effects in both short and long SOAs. Thus, future studies should consider to include both compound characters and single characters as primes in one study in order to dissociate radical and lexical level priming effects.

Nevertheless, the present study not only consolidated the past studies of robust semantic priming effect on Chinese character recognition, but it also expands prior findings showing a graded priming effect of semantic relatedness between semantic radicals and the characters containing them. The findings of the present study provide empirical support for the psycholexical space.

## Data Availability Statement

The raw data supporting the conclusions of this article will be made available by the authors, without undue reservation.

## Ethics Statement

The studies involving human participants were reviewed and approved by Hang Zhou Normal University. The patients/participants provided their written informed consent to participate in this study. Written informed consent was obtained from the individual(s) for the publication of any potentially identifiable images or data included in this article.

## Author Contributions

XT conceived and designed this study. MX collected the data under the guidance of XT and JZ. XT, MX, and LY conducted the data analysis and interpreted the results. XT and MX draft the manuscript. All authors reviewed and approved the final manuscript.

## Conflict of Interest

The authors declare that the research was conducted in the absence of any commercial or financial relationships that could be construed as a potential conflict of interest.
